# Immunotherapy‐Related Cystitis Induced by Nivolumab for Advanced Gastric Cancer: A Case Report

**DOI:** 10.1155/criu/4082092

**Published:** 2026-04-15

**Authors:** Kentaro Arinami, Kozue Ito, Go Hasegawa, Yurie Takizawa, Yohei Ikeda, Takeshi Suda, Noboru Hara, Tsutomu Nishiyama

**Affiliations:** ^1^ Department of Urology, Uonuma Institute of Community Medicine, Niigata University Medical and Dental Hospital, Minamiuonuma, Niigata, Japan, niigata-u.ac.jp; ^2^ Department of Pathology, Uonuma Institute of Community Medicine, Niigata University Medical and Dental Hospital, Minamiuonuma, Niigata, Japan, niigata-u.ac.jp; ^3^ Department of Diagnostic Radiology, Uonuma Institute of Community Medicine, Niigata University Medical and Dental Hospital, Minamiuonuma, Niigata, Japan, niigata-u.ac.jp; ^4^ Department of Gastroenterology and Hepatology, Uonuma Institute of Community Medicine, Niigata University Medical and Dental Hospital, Minamiuonuma, Niigata, Japan, niigata-u.ac.jp

**Keywords:** advanced gastric cancer, corticosteroid treatment, immunotherapy-related cystitis, nivolumab

## Abstract

A 70‐year‐old man received treatment for advanced gastric cancer involving capecitabine, oxaliplatin, and nivolumab therapy. Following four courses of this treatment, he developed severe urethralgia and hematopyuria. Urine bacterial culture was negative. Cystoscopy revealed readily bleeding and edematous, reddish mucosa. Computed tomography identified edematous changes in the wall of the bladder, but no lesions were noted in the upper urinary tract. The bladder wall was biopsied, and pathological findings indicated erosion and hemorrhage of the bladder mucosa, accompanied by lymphocytic infiltration. Based on the overall clinical presentation, immunotherapy‐associated cystitis induced by nivolumab treatment for advanced gastric cancer was suspected. He began taking prednisolone at a dosage of 25 mg per day, which was gradually tapered over time, and the urinary symptoms markedly improved.

## 1. Introduction

Immune‐related adverse events (irAEs) may arise during treatment with immune checkpoint inhibitors (ICIs), potentially necessitating steroid therapy. [1] However, nonbacterial cystitis resulting from ICI use is uncommon. Here, we report nonbacterial cystitis as an irAE in a patient receiving nivolumab for advanced gastric cancer.

## 2. Case Presentation

**Figure 1:**
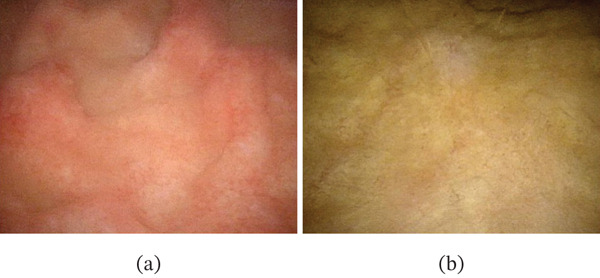
Cystoscopy findings. (a) Cystoscopy performed at the onset of irritative bladder symptoms revealed edematous and erythematous mucosa. (b) After treatment with prednisolone, cystoscopy showed no abnormal findings.

**Figure 2:**
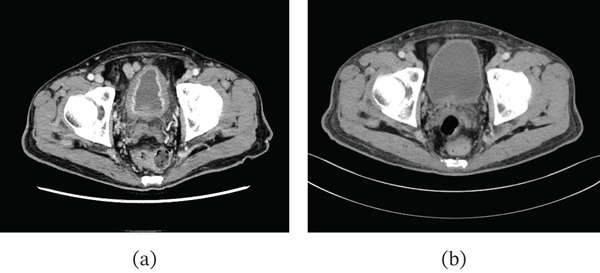
CT findings. (a) CT at the onset of irritative bladder symptoms revealed edematous changes in the bladder wall. (b) After treatment with prednisolone, CT findings showed significant improvement in the bladder wall.

**Figure 3:**
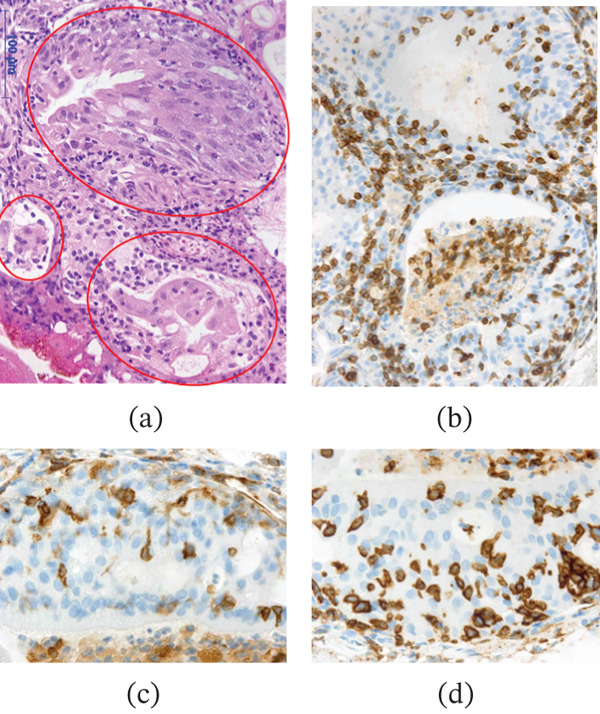
Pathological findings. The remaining urothelial epithelium (indicated by circles) exhibited significant polarity disruption and lymphocytic infiltration, accompanied by degeneration and atrophy. (a) Lymphocyte infiltration predominantly comprised T lymphocytes, including (b) CD3‐, (c) CD4‐, and (d) CD8‐positive cells, which infiltrated the urothelial epithelium. The immunohistochemical findings, showing a predominance of CD8‐positive over CD4‐positive T cells in this case, were nonspecific but suggested irAE cystitis.

A 70‐year‐old man received a first‐line treatment for advanced gastric cancer involving capecitabine, oxaliplatin, and nivolumab therapy. Following four courses of this treatment, he developed severe urethralgia and hematopyuria. Urine bacterial culture was negative. Cystoscopy revealed edematous, reddish mucosa. (Figure 1a) Computed tomography (CT) identified edematous changes in the bladder wall, but no lesions were noted in the upper urinary tract. (Figure 2a) The wall of the bladder was biopsied, with pathological findings indicating erosion and hemorrhage of the bladder mucosa, accompanied by lymphocytic infiltration. (Figure 3) Immunohistochemical staining demonstrated the presence of CD3‐ and CD5‐positive T cells, along with CD4‐ and CD8‐positive cells, which were nonspecific, but they suggested that the cystitis may be irAE. As the symptoms were worsening daily, the patient was diagnosed with immunotherapy‐related cystitis as irAE resulting from nivolumab treatment for advanced gastric cancer. Nivolumab was discontinued, and he began taking prednisolone, a corticosteroid, at a dosage of 25 mg per day, which was gradually tapered over time. This treatment led to markedly improved urinary symptoms, with both cystoscopy (Figure 1b) and CT (Figure 2b) showing significant improvement.

## 3. Discussion

Biologics known as ICIs include monoclonal antibodies that target PD‐1, PD‐L1, and CTLA‐4, which are used in cancer immunotherapy. [2] Inhibiting these molecules, which suppress immune cell activation, enables the activated immune cells to attack and destroy tumor cells, thereby suppressing tumor growth. As a result of their mechanism of action, ICIs can cause irAEs in various tissues. [1, 2] The frequency of irAEs depends on the drug used, treatment duration, dosage, and patient‐specific risk factors; however, the timing of onset is often influenced by the affected organ system. irAEs can affect multiple organ systems, with common irAEs involving the skin, gastrointestinal tract, liver, endocrine organs, and lungs. The onset of irAEs typically occurs within weeks to months after initiating ICI therapy, but can also occur within days of the initial dose. Furthermore, delayed toxicity may arise even after the discontinuation of ICI therapy.

Reports of irAEs related to the bladder or urinary tract are rare. [3, 4] Currently, no consensus criteria exist regarding the grading of immune‐mediated cystitis. The primary symptoms of this cystitis include urinary irritation, such as urgency, pain, frequent urination, and hematuria. In the present patient, nivolumab was administered to treat gastric cancer, after which refractory bladder irritation symptoms developed. Based on the overall clinical presentation, immunotherapy‐associated cystitis induced by nivolumab treatment for advanced gastric cancer was suspected in this case. Wang et al. identified sterile urine cultures and a history of ICI administration as key factors in diagnosing irAE ureteritis and cystitis. [4] They also reported that the time to onset of irAE ureteritis and cystitis exhibited a wide temporal distribution, ranging from 28 to 442 days. This patient developed symptoms of bladder irritation 12 weeks after initiating first‐line treatment for advanced gastric cancer, which included capecitabine, oxaliplatin, and nivolumab. There are almost no reported cases of drug‐induced cystitis caused by capecitabine or oxaliplatin. Therefore, based on the overall clinical presentation, cystitis associated with immunotherapy, induced by nivolumab treatment for advanced gastric cancer, was suspected in this case.

Clinically, the presence of these symptoms is crucial for diagnosing immune‐mediated cystitis and promoting prompt consideration of the side effects of immunotherapy. Furthermore, the primary feature observed during cystoscopy was a reddened, diffusely edematous bladder mucosa, indicating a widespread inflammatory reaction. (Figure 1) There were no signs of a focal tumor, with most findings suggesting diffuse mucosal changes. Histopathological examination revealed urothelial infiltration into the stroma, but no atypia of the urothelium was observed. The stroma was infiltrated by lymphocytes, plasma cells, and eosinophils, with localized lymphoid follicle formation being noted. The inflammatory infiltrate stimulated capillary proliferation and caused edema in bladder mucosal tissue.

Activation of T cells by ICIs is considered to play a role in cancer‐related immunity and also a cause of irAEs. CD8‐positive T cells play a crucial role in cancer immunology and are also significant in the development of irAEs. [5, 6] Immunohistochemical staining revealed a mixture of T lymphocytes positive for CD3 and CD4 markers. Recently, it has been reported that irAE target organs exhibit a higher infiltration of CD8‐positive T lymphocytes compared with CD4‐positive T lymphocytes. [5, 6] The immunohistochemical findings, showing a predominance of CD8‐positive over CD4‐positive T cells in this case, were nonspecific but suggested irAE cystitis.

Immune‐related cystitis is an immune‐mediated side effect typically caused by ICIs, although the exact mechanism remains unclear. Immunotherapy can induce a cytokine storm, particularly through the activation of CD8‐positive T cells, which may trigger an excessive immune response in the bladder, resulting in cystitis. It has been suggested that ICIs activate T cells by inhibiting the PD‐1/PD‐L1 pathway, resulting in attacks not only on tumor cells but also on normal bladder epithelial cells. [7] Some previous authors hypothesized that immune‐related cystitis may result from an autoimmune response targeting antigens, such as BP180 and integrins *α*6 and *β*4. [8] In this case, cystoscopy revealed erythematous and edematous mucosa, CT images showed thickening of the bladder wall, and pathological findings of inflammatory infiltration made it difficult to clearly distinguish classic irAE‐related cystitis from the other immune‐related cystitis such as Hunner‐type interstitial cystitis. Wang et al. reported a single‐center study involving 12 cases of ICI‐related ureteritis and cystitis, noting that the incidence is less than 1%. They also observed that the disease is characterized by negative urine cultures and a history of ICI administration. [4] Additionally, rapid improvement with corticosteroid treatment was an important feature for confirming the diagnosis of ICI‐related ureteritis and cystitis. The diagnosis and treatment of irAE cystitis, as described by Wang et al., involve corticosteroid therapy guided by a negative urine culture and a history of ICI administration, followed by an assessment of treatment response. Steroid treatment led to a marked improvement in urinary symptoms, as well as in both cystoscopy (Figure 1b) and CT imaging (Figure 2b) findings in this patient.

For treatment, corticosteroids provide rapid symptomatic relief; however, caution is necessary on tapering them, as this may lead to the recurrence of irAEs, worsening of the disease, or development of novel complications. [1, 2] What constitutes optimal treatment, including the appropriate steroid dosage and duration, remains a subject of debate. Regarding immune‐mediated cystitis, most patients respond well to steroids. However, Fukunaga et al. recently reported a case of steroid‐resistant cystitis developing during nivolumab treatment for lung cancer. [9] For steroid‐resistant immune‐related cystitis, the use of infliximab, an antihuman TNF‐*α* monoclonal antibody, as well as JAK‐STAT inhibitors, has been reported and should be considered. [4]

Discontinuation of ICIs may be necessary for some patients. In the present case, the patient discontinued nivolumab and resumed treatment with capecitabine and oxaliplatin.

## 4. Conclusion

We reported a gastric cancer patient who developed intractable bladder irritation symptoms, including hematopyuria, following treatment with nivolumab. Immune‐mediated cystitis presents with nonspecific symptoms and lacks standardized diagnostic criteria. Early treatment with corticosteroids is recommended to help prevent the overuse of antibiotics.

## Author Contributions

Kentaro Arinami: writing—original draft and writing. Kozue Ito: writing—review and editing. Yurie Takizawa: writing—review and editing. Go Hasegawa: writing—review and editing. Yohei Ikeda: writing—review and editing. Takeshi Suda: writing—review and editing. Noboru Hara: writing—review and editing. Tsutomu Nishiyama: writing—original draft and writing—review and editing.

## Funding

No funding was received for this manuscript.

## Disclosure

All authors have read and approved the final version of the manuscript. Tsutomu Nishiyama, the corresponding author, had full access to all of the data in this study and takes complete responsibility for the integrity of the data and the accuracy of the data analysis.

## Ethics Statement

The authors have nothing to report.

## Consent

Written informed consent was obtained from the patient for the publication of this case report and accompanying images.

## Conflicts of Interest

The authors declare no conflicts of interest.

## Data Availability

Data sharing is not applicable to this article as no datasets were generated or analyzed during the current study.
